# Increased Entropic Brain Dynamics during DeepDream-Induced Altered Perceptual Phenomenology

**DOI:** 10.3390/e23070839

**Published:** 2021-06-30

**Authors:** Antonino Greco, Giuseppe Gallitto, Marco D’Alessandro, Clara Rastelli

**Affiliations:** 1 Department of Psychology and Cognitive Science, University of Trento, 38068 Rovereto, Italy; clara.rastelli@unitn.it; 2 Centre for Integrative Neuroscience, University of Tübingen, 72076 Tübingen, Germany; 3 MEG Center, University of Tübingen, 72076 Tübingen, Germany; 4 Department of Neurology, University Hospital Essen, 45147 Essen, Germany; giuseppe.gallitto@uk-essen.de; 5 Institute of Cognitive Sciences and Technologies, National Research Council, 00185 Rome, Italy; marco.dal92@gmail.com

**Keywords:** psychedelics, DeepDream, entropic brain dynamics, altered perception, deep neural networks, EEG

## Abstract

In recent years, the use of psychedelic drugs to study brain dynamics has flourished due to the unique opportunity they offer to investigate the neural mechanisms of conscious perception. Unfortunately, there are many difficulties to conduct experiments on pharmacologically-induced hallucinations, especially regarding ethical and legal issues. In addition, it is difficult to isolate the neural effects of psychedelic states from other physiological effects elicited by the drug ingestion. Here, we used the DeepDream algorithm to create visual stimuli that mimic the perception of hallucinatory states. Participants were first exposed to a regular video, followed by its modified version, while recording electroencephalography (EEG). Results showed that the frontal region’s activity was characterized by a higher entropy and lower complexity during the modified video, with respect to the regular one, at different time scales. Moreover, we found an increased undirected connectivity and a greater level of entropy in functional connectivity networks elicited by the modified video. These findings suggest that DeepDream and psychedelic drugs induced similar altered brain patterns and demonstrate the potential of adopting this method to study altered perceptual phenomenology in neuroimaging research.

## 1. Introduction

In recent years, there has been a renewed interest in the use of psychedelics drugs (e.g., LSD, psilocybin) to understand the neural dynamics of conscious perception, in the neuroscientific community. Many studies attempted to investigate the neural mechanisms that cause altered conscious perception [1,2,3,4,5] as well as examine the potential use of psychedelics in psychotherapy [6,7,8]. For instance, Sarasso et al. [9] reported ketamine-induced complex cortical activation patterns similar to wakefulness states. Using psilocybin, Petri et al. [10] found that brain functional patterns were characterized by several transient low-stability structures, and Tagliazucchi et al. [11] reported a larger repertoire of brain dynamical states. Similarly, Schartner et al. [12] showed evidence of increased entropy in the magnetoencephalography (MEG) signal using LSD, psilocybin and ketamine. Moreover, Roseman et al. [5] and Barnett et al. [13] reported an increased functional connectivity triggered by the ingestion of psychedelic drugs that could in principle lead to an increase in the entropy of the brain dynamics. These findings led to the recent conceptualization of the entropic brain hypothesis [3], which explains the altered states of consciousness induced by psychedelics in terms of higher entropy of the brain’s activity and functional connectivity. However, the use of psychedelic substances in scientific investigations is hindered by ethical and legal issues, since these drugs are prohibited in many countries by law and obtaining ethical approvements for their scientific usage might not be trivial. Moreover, the systemic physiological effects of psychedelic compounds posit a critical experimental challenge due to the difficulty in isolating them from cognitive effects of interest. To overcome these problems, Suzuki et al. [14] proposed a methodology, called the Hallucination Machine, that combines deep convolutional neural networks (CNNs) and panoramic videos viewed immersively through virtual reality (VR) to simulate biologically plausible visual hallucinations. They found that this methodology induced perceptual phenomenology qualitatively similar to psychedelics, albeit did not present the temporal distortion usually accompanying altered states. Here, we capitalized on the work of Suzuki et al. [14], which explored only the behavioral effects of simulated hallucinations, to investigate the neural effects of presenting artificial hallucinations. To the best of our knowledge, this is the first study exploring brain dynamics associated with artificially-induced altered perceptual phenomenology. We exposed participants to two video clips, one regular and one modified by DeepDream [15], using the same algorithm implemented by Suzuki et al. [14] to create simulated hallucinations, while recording electroencephalography (EEG). We tested whether DeepDream-induced brain dynamics are characterized by a more chaotic regime with respect to regular visual perception, by comparing entropy and statistical complexity measures on the EEG signal in the time domain and recently developed entropy measures for functional connectivity networks.

## 2. Materials and Methods

### 2.1. Participants

Twenty volunteers (12 females, mean age 26.4, range 22–31) participated in this study. All were right-handed with normal or corrected-to-normal vision and normal hearing, had no history of neurological disorders and were not taking any neurological medications. All participants gave informed written consent. The study was conducted in accordance with the Declaration of Helsinki and approved by the University of Trento Ethics Committee.

### 2.2. Stimuli and Procedure

**Figure 1:**
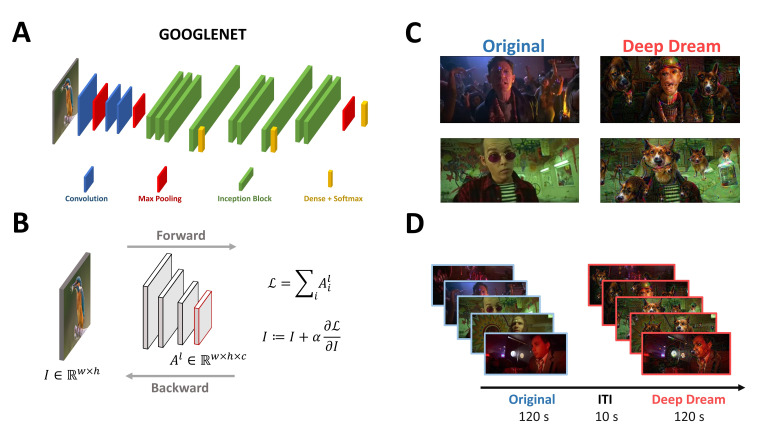
(**A**) Visual representation of the architecture of GoogleNet. (**B**) General description of the DeepDream algorithm. (**C**) Examples of frames from the OR and DD videos. (**D**) Schematic representation of the time course of the experiment.

Participants were sitting in a dimly-lit booth at a distance of 1 m from the CRT monitor (22.5 in VIEWPixx; resolution: 1920 × 968 pixels; refresh rate: 120 Hz; screen width: 50 cm). The experiment consisted of watching two video clips (24.4° × 16.3° visual angle, 720 × 304 resolution, 18 fps) with a duration of 120 s each and an interval of 10 s between the clips (Figure 1D). One video clip was extracted from a movie and is referred to as the original condition (OR), while the other video clip was a modification of the original one using DeepDream and it is referred to as the DeepDream condition (DD). We opted to maintain a fixed order of the conditions for two reasons. First, from a brief pilot we conducted, we observed that the exposure to the DD condition seemed to be a “strong” experience for participants, so we were concerned that by flipping the conditions there could be some tracing effects of DD over the OR condition. Second, both in the pilot and in the experiment, many participants did not recognize the DD video as the modified version of the OR one, therefore we estimated the chances of possible learning effects taking place being low. DeepDream is an algorithm that alters and enhances patterns in images through a process that can be conceived as “algorithmic pareidolia” and relies on a pre-trained deep convolutional neural network (CNN). It works by passing an input image I with width (w) and height (h) through the pre-trained CNN up to a selected layer $A^{l}$, with the objective to maximize the layer activation (Figure 1B). To do so, instead of optimizing the parameters of the network to reach the goal, as in the classic approach, it alters the input image by adding the gradients of this objective function ℒ (i.e., the selected layer activation) computed with respect to the input image. This procedure is called gradient ascent because it leads to the maximization of the objective function. Since DeepDream was conceived for static images, we followed Suzuki et al. [14] for adapting the algorithm to videos using optical flow to stabilize the optimization process and reduce variability of the generated hallucinatory patterns across frames (Figure 1C). The DD video was generated by selecting a higher layer (inception_4d/pool) of the GoogleNet CNN [15,16] (Figure 1A), and setting all the hyperparameters similarly to [14] (octaves = 3, octave scale = 1.8, iterations = 16, jitter = 32, zoom = 1, step size = 1.5, flow threshold = 6, blending ratio for optical flow = 0.9, blending ratio for background = 0.1).

### 2.3. EEG Acquisition and Preprocessing

EEG data were recorded from a standard 10-5 system with 27 Ag/AgCl electrodes cap (EasyCap, Brain Products, Germany) at a sampling rate of 1 kHz. Impedance was kept below 10 kΩ for all channels. AFz was used as the ground and the right mastoid was used as reference. Electrodes were positioned at the following scalp sites: Fpz, Fz, F3, F4, F7, F8, F9, F10, FC5, FC6, T7, C3, Cz, C4, T8, CP5, CP6, P7, P3, Pz, P4, P8, PO7, PO8, O1, Oz, and O2. All preprocessing steps were conducted using EEGLAB [17]. Spherical interpolation was carried out on individual bad channels with the criterion that a channel correlated less than 0.85 on average compared to its neighbors [18] and with the assistance of visual inspection (average number of interpolated channels: 1.4, range: 0–6). Data were filtered with a high-pass at 0.1 Hz and a low-pass at 80 Hz, using a Butterworth IIR filter with model order 2. CleanLine [19] with default parameters was used to remove line noise at 50 Hz and its harmonics up to 200 Hz. After this step, data were re-referenced to a common average reference and stereotyped artifacts, including blinks, eye movements and muscle artifacts were deleted via independent component analysis (ICA) via the extended infomax algorithm [20].The average number of removed independent components was 11.95 (±2.66 SD), following a rejection strategy based on ICLabel [21] and visual inspection. Finally, data were converted to FieldTrip [22] format for subsequent analyses.

### 2.4. Entropy and Complexity Analysis of the EEG Signal

Data were firstly analyzed in terms of entropy and complexity of the EEG signal. For the entropy measure, we relied on a combination of several improvements of the classical Shannon Entropy (H) measure that are well suited for EEG data. First, we used Permutation Entropy (PE) [23] because it can better characterize noisy signals such as the EEG [24]. Consider the time-series process $\left\{x_{t}\right\}$, t = 1, …, T, encoding the EEG signal at each channel. For the computation of PE, we consider vectors $x_{1}^{*},\;x_{2}^{*},\ldots ,\;x_{k}^{*}$ obtained by embedding the time-series $\left\{x_{t}\right\}$ into m-dimensional space, such that:

(1) $$x_{k}^{*}=\left(x_{k},x_{k+\tau },\ldots ,\;x_{k+\left(m-1\right)\tau }\right),\;k=1,\;2,\;\ldots ,\;T-\left(m-1\right)\tau$$

where *m* and *τ* denote the embedding dimension and time delay, respectively. Then, the elements of $x_{k}^{*}$ are arranged in ascending order such that $\left\{x_{k+\left(i_{1}-1\right)\tau }\;\leq x_{k+\left(i_{2}-1\right)\tau }\;\leq \ldots \;\leq \;x_{k+\left(i_{m}-1\right)\tau }\right\}$. In the m dimensional space, each ordered vector $x_{k}^{*}$ is mapped to a single motif out of m! possible ordinal patterns $\pi_{i}$. For a permutation with number $\pi_{i}$, let $f\left(\pi_{i}\right)$ denote the frequency of the *i*-th permutation in the time series. Then the probability of each ordinal pattern can be defined as:

(2) $$p\left(\pi_{i}\right)=\frac{f\left(\pi_{i}\right)}{\sum_{i=1}^{m!}f\left(\pi_{i}\right)}$$

Thus, the computation of PE is just the H applied to the probability function of the ordinal patterns:

(3) $$PE\left(p\right)=-\overset{m!}{\underset{i=1}{\sum }}p\left(\pi_{i}\right)\log\left(p\left(\pi_{i}\right)\right)$$

To obtain a normalized PE value between 0 and 1, we divided this quantity by log(m!), which is the entropy of the uniform distribution ($p_{u}$) with m! bins. In this study, we set τ = 1 and m = 5 because we aimed to obtain a good balance between having a reasonable number of ordinal patterns (120) and the demand of computational resources. Although PE is a robust measure of nonlinear time-series, its main limitation is its inability to differentiate between distinct forms of a certain ordinal pattern and the sensitivity of distinguishing background noise modes. This is because PE, through the symbolization process, only retains the ordinal structure when extracting the ordinal patterns. To overcome this limitation and include important information when retrieving motifs such as the signal’s amplitude, Fadlallah et al. [25] proposed a weighted version of the symbolization process of PE. In this version, the relative frequencies for the *i*-th motif are weighted according to:

(4) $$p_{\omega }\left(\pi_{i}\right)=\frac{\sum_{b=1}^{B}f\left(\pi_{i}^{b}\right)\;\cdot \;\omega_{b}}{\sum_{i=1}^{m!}\sum_{b=1}^{B}f\left(\pi_{i}^{b}\right)\;\cdot \;\omega_{b}}$$

(5) $$\omega_{b}=\frac{1}{m}\overset{m}{\underset{n=1}{\sum }}{\left(x_{\left(b+\tau \left(n-1\right)\right)}-{\bar{x}}_{b}^{*}\right)}^{2}$$

where B is the number of the possible patterns in the same motif and ${\bar{x}}_{b}^{*}$ denotes the arithmetic mean of the embedded vector $x_{b}^{*}$. Thus, the weighted permutation entropy (WPE) is defined as:

(6) $$WPE\left(p_{\omega }\right)=-\overset{m!}{\underset{i=1}{\sum }}p_{\omega }\left(\pi_{i}\right)\log\left(p_{\omega }\left(\pi_{i}\right)\right)$$

Finally, to obtain a measure of entropy spanning across multiple time scales, we applied a coarse graining procedure to the time-series by averaging consecutive data points according to the time scale parameter θ, and then we computed the WPE on the resulting signal. We defined this measure as multiscale weighted permutation entropy (MWPE). Each element $y_{k}^{\theta }$ of the coarse-grained time series is calculated as follows:

(7) $$y_{k}^{\theta }=\frac{1}{\theta }\overset{k\theta }{\underset{i=\left(k-1\right)\theta +1}{\sum }}x_{i},\;1\leq k\leq \frac{T}{\theta }$$

The length of each coarse-grained time series is T/θ and the coarse-grained time-series with θ = 1 is the original time-series. Here, we set θ from 1 to 20, thus ranging from a time scale of 1 ms (the signal was sampled at 1 kHz) to 20 ms. We decided to analyze the data up to θ = 20 because the resulting coarse-grained signal had a reasonable amount of data points for computing the entropy measure (i.e., 6000). For the complexity measure, we relied on the Jensen–Shannon complexity (JSC) introduced by Rosso et al. [26]. JSC is a statistical measure of the complexity of a signal based on the product of the Jensen–Shannon divergence (JSD), a measure of the signal’s entropy and a normalization constant as follows:

(8) $$JSC\left(p_{\omega }\right)=-2\frac{H\left(\frac{p_{\omega }+p_{u}}{2}\right)-\frac{1}{2}H\left(p_{\omega }\right)-\frac{1}{2}H\left(p_{u}\right)}{\frac{m!+1}{m!}\log\left(m!+1\right)-2\log\left(m!\right)+\log\left(m!\right)}\psi \left(p_{\omega }\right)$$

where ψ in this study is the WPE. The quantity in the denominator is used as a normalization constant for the JSD. The JSD can be interpreted as the distance between the distribution of ordinal patterns p(π) and the uniform distribution $p_{u}$. JSC has a parabolic shape when plotted against the PE and, crucially, can hold multiple values for a fixed amount of entropy. Thus, JSC allows to differentiate between regimes that are highly deterministic or highly stochastic and everything in-between [26]. We decided to use this measure since it can provide a different point of view about the irregularity of the signal with respect to PE, providing more information about the correlational structures rather than chaoticity [27]. Since we computed this measure across multiple time scales and with WPE, we defined this measure as multiscale weighted Jensen–Shannon complexity (MWJSC). Both MWPE and MWJSC were applied to each channel individually, for each participant and condition. To combine these two measures of the irregularity of the signal, we also used a tool known in the literature as the entropy-complexity plane [28,29] to both analyze and visualize the data along these two dimensions. The entropy-complexity plane allowed us to investigate the data in a complementary way, by looking simultaneously at these two aspects of the signal. Specifically, since we also had the time scale dimension, we defined this tool as the entropy-complexity hyperplane (ECH). Our decision to apply MWPE and MWJSC measures was two-fold. Firstly, these are improved versions of more classical measures like PE and JSC, and secondly, the interpretation of these measures is not different from the classical ones, allowing a straightforward comparison with previous studies in the field. For completeness, we also analyzed the data using the multiscale versions of PE and JSC, namely multiscale permutation entropy (MPE) and multiscale Jensen–Shannon complexity (MJSC). Furthermore, following a suggestion from one of our reviewers, we also analyzed the data using another common measure of entropy called Lempel–Ziv complexity (LZC) [30]. LZC also uses a symbolization procedure in its algorithm, so we used both the median binarization procedure (i.e., every value of the signal below the median becomes 0 and above is 1) and the same embedding procedure for the computation of PE with the same parameters described above. Thus, we ended up with, respectively, median LZC (mLZC) and permutation LZC (pLZC).

### 2.5. Functional Connectivity Networks and Geodesic Entropy Estimation

Functional connectivity networks were estimated from the EEG signal using a phase-based approach. First, data from each participant and condition were split into 120 non-overlapping windows of 1 s each. Then, we applied spectral analysis (using the mtmfft function in FieldTrip) to each window using an Hanning taper to compute the sensor cross-spectral density in three different frequency bands such as alpha (7–13 Hz), beta (13–25 Hz) and gamma (25–45 Hz). After this step, functional connectivity was estimated by means of the weighted phase lag index (WPLI) and results were averaged across windows and within each frequency band. To estimate the significance of the WPLI values, we computed 200 surrogate signals by taking the actual signal, applied the fast Fourier transform (FFT), randomized the phase and then took the inverse FFT. We applied the same pipeline for obtaining WPLI values to the surrogate data and took the 95th percentile of the surrogates’ distribution as a threshold for masking the actual connectivity values. Finally, we ended up with a binarized connectivity network for each participant, condition and frequency band. Thus, we defined the global functional connectivity (GFC) measure as the sum of all significant connections in a network. Furthermore, for each network, we computed the recently developed geodesic entropy (GE) [31] measure of network entropy. In its essence, GE is able to quantify the information due to the intrinsic configuration of network architecture, characterizing the interdependence of influences in the network [31]. In the network science literature, a network is identified by a set of nodes V and a set of links E. A geodesic D(i,j) is defined as the shortest path between two nodes (i.e., the minimal number of consecutive links connecting two nodes). A probability mass function $p_{i}\left(r\right)$ is defined by finding a node in the neighborhood ratio r of the node i, that is, the probability that one selects randomly a node j from the set of the remaining nodes set $\bar{v}=\{j|j\;\in V\backslash \left\{i\right\}\}$ with the geodesic distance D(i,j)=r. Thus, $p_{i}\left(r\right)$ is defined as:

(9) $$p_{i}\left(r\right)=\frac{1}{N-1}\underset{j\in \bar{v}}{\sum }\delta_{D\left(i,j\right),r}$$

where r can take values according to the interval $1\leq r\leq r_{max}$ with $r_{max}=max_{j\in \bar{v}}\left(D\left(i,j\right)\right)$ and *N* is the number of nodes. The GE relative to each node is given by the H of the probability distribution computed on its geodesics, while the GE of the entire network can be computed by averaging nodes’ GE (AGE).

### 2.6. Statistical Analysis

Statistical analysis for MWPE and MWJSC was assessed by means of cluster corrected non-parametric permutation two-sided paired *t*-tests [32] with 10,000 randomizations and α=0.05 on selected region of interest (ROI) and time scales. The selected ROIs were frontal (Fpz, Fz, F3, F4, F7, F8, F9, F10), temporo-parietal (FC5, FC6, T7, C3, Cz, C4, T8, CP5, CP6, P3, Pz, P4) and occipital (P8, PO7, PO8, P7, O1, Oz, O2). Since we needed to define a neighbor structure for the cluster correction procedure [32], it is non-trivial to do it in our ROI space compared to more trivial dimensions like frequency bands or time scales. Therefore, we decided to create a neighbor structure by connecting only ROIs which are ideally closer to each other, leading to a neighbor structure with the temporo-parietal ROI connected with the other two (frontal and occipital), and these two connections being the only ones in the structure. Thus, clusters were computed across the whole space-time volume. Effect size was assessed using Cohen’s d [33] and reported the mean and max values for the significant clusters. For the ECH, we used a multivariate pattern analysis (MVPA) approach [34] by using linear discriminant analysis (LDA) to discriminate between the two conditions using MWPE and MWJSC as features in the selected ROIs. We used leave-one-subject-out (LOSO) cross-validation, area under curve (AUC) as metric performance (and as a measure of effect size) and statistical significance was assessed against the estimated chance level by performing the same MVPA analysis with permuted labels, also using cluster corrected non-parametric permutation two-sided paired *t*-tests. For the AGE and GFC, we used the same cluster corrected procedure to compare DD and OR conditions, with the difference being that in this case the clusters were computed across the frequency bands’ dimension.

## 3. Results

### 3.1. Entropy and Complexity

**Figure 2:**
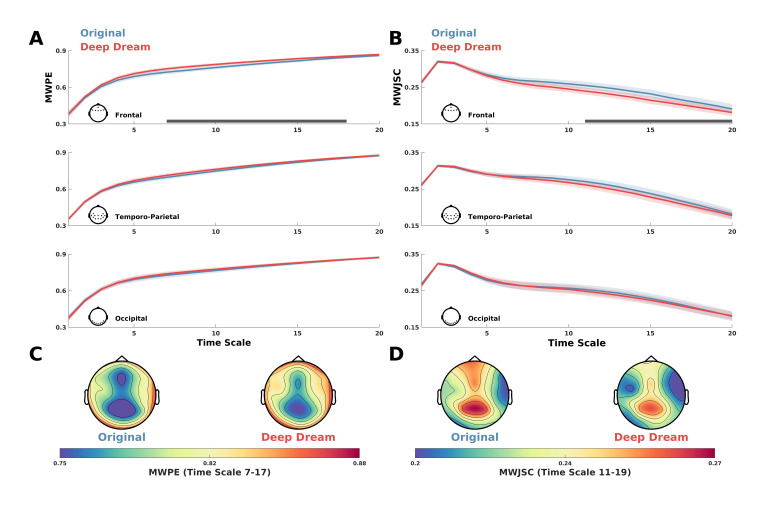
(**A**) MWPE values for DD and OR along the time scales in the frontal, temporo-parietal and occipital ROI. Shaded areas represent standard error of the mean. Gray lines indicate statistical significance (*p* < 0.05, cluster corrected). (**B**) MWJSC values for DD and OR along the time scales in the frontal, temporo-parietal and occipital ROI. Shaded areas represent standard error of the mean. Gray lines indicate statistical significance (*p* < 0.05, cluster corrected). (**C**) Topographic maps depicting the MWPE of DD and OR in the significant time-scale range 7–17. (**D**) Topographic maps depicting the MWJSC of DD and OR in the significant time scale-range 11–19.

**Figure 3:**
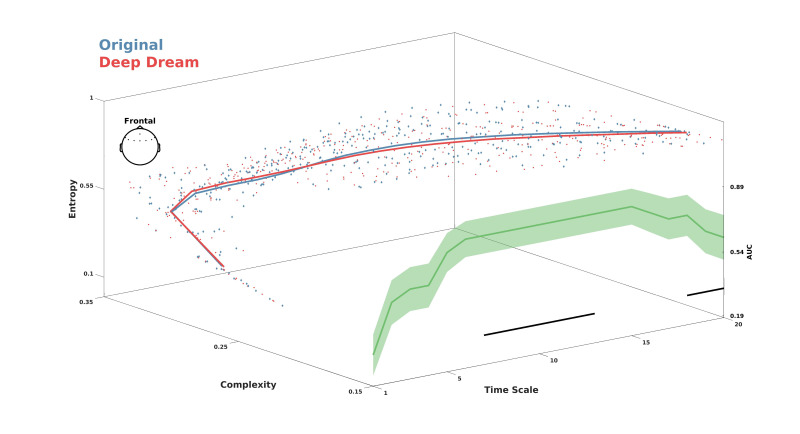
Entropy complexity hyperplane (ECH) showing MWPE and MWJSC values conjunctively across the time scales. Red and blue lines represent average values for DD and OR. Colored dots are participants’ values. Green line indicates the performance of the LDA classifier. Shaded areas represent standard error of the mean. Black lines indicate statistical significance (*p* < 0.05, cluster corrected).

MWPE analysis of the signal revealed a significant higher entropy level of the DD video with respect to OR in the frontal ROI between time-scale range 7–17 (Figure 2A; p = 0.036, $d_{mean}$ = 0.60, $d_{max}$= 0.71). In addition, a general spatial trend of increased entropy of DD over OR was observed by looking at the topographic maps of the sensors’ MWPE values (Figure 2C). Conversely, MWJSC analysis revealed a significant lower complexity level of DD with respect to OR, also in the frontal ROI but along the time-scale range 11–19 (Figure 2B; p = 0.043, $d_{mean}$ = 0.58, $d_{max}$ = 0.71). From the topographic maps, we also observed a trend of decreased complexity of DD compared to OR over the parietal channels, but this effect did not surpass statistical significance (Figure 2B–D). MPE analysis also revealed the same pattern of MWPE, with higher entropy over frontal regions along the time scale range 6–16 in DD with respect to OR (Figure S1A–C; p = 0.033, $d_{mean}$ = 0.59, $d_{max}$ = 0.63), although the effect size was reduced with respect to the MWPE analysis. This was also the same for MJSC, showing decreased statistical complexity of DD with respect to OR in frontal regions along the time scale range 7–17 (Figure S1B–D; p = 0.027, $d_{mean}\;$= 0.59, $d_{max}\;$= 0.64), but again with less effect size compared to MWJSC. Regarding LZC measures, mLZC did not showed any significant difference and seemed almost identical (from visually inspecting the results in Figure S2A) between the two conditions, while pLZC showed an increased trend of DD compared to OR over frontal regions in time-scale range 9–14 similarly to MWPE and MPE, but this effect did not surpass statistical significance (Figure S2B). MVPA analysis on the ECH representation confirmed the two previous findings by showing a significant performance in the time scale clusters 6–11 ($p=0.008,\;AUC_{mean}\;$= 0.80) and 18–20 (p = 0.017, $AUC_{mean}$ = 0.69). Moreover, ECH examination qualitatively revealed how entropy and complexity conjunctively differentiate the two conditions from lower to higher time scales (Figure 3).

### 3.2. Global Functional Connectivity and Geodesic Entropy

**Figure 4:**
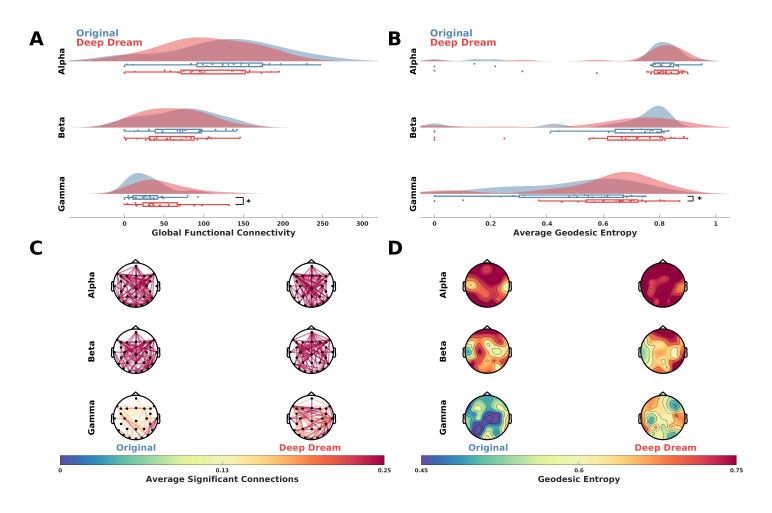
(**A**) Raincloud plots showing GFC values in the alpha, beta and gamma bands between DD and OR. Asterisk indicates statistical significance (*p* < 0.05). (**B**) Raincloud plots showing AGE values in the alpha, beta and gamma bands between DD and OR. Asterisk indicates statistical significance (*p <* 0.05). (**C**) Functional connectivity networks in the alpha, beta and gamma bands represented as topographic connectivity plots. (**D**) Topographic maps showing sensor GE in DD and OR in the alpha, beta and gamma bands.

Functional connectivity analysis showed a higher GFC value in DD with respect to OR in the gamma band (Figure 4A; *p =* 0.016, d = 0.59). This is also depicted in Figure 4C, where the average significant connections across participants are plotted as connectivity topographic maps. Moreover, in the gamma band the AGE was higher in DD with respect to OR (Figure 4B; *p* = 0.041, d = 0.51). Topographic representations of sensor GE showed this effect was largely driven by fronto-parietal sensors (Figure 4D).

## 4. Discussion

In this paper, we investigated the brain dynamics related to artificially-induced altered perception. We used DeepDream to synthesize a video possessing perceptual characteristics similar to perceptual experiences elicited by psychedelic drugs. By comparing the EEG signal of the DD video and the OR video, we found an increase in the entropy level and a decrease in the statistical complexity level of the DD condition compared to the OR condition in the frontal regions. This finding is comparable to some studies in the literature, in which a strong modulation of frontal areas induced by Ayahuasca ingestion was reported [35,36]. Surprisingly, these differences in entropy and complexity of the signal were located at different time scales. Entropy was higher in lower time scales, while complexity was lower at higher time scales, as also shown in the ECH (Figure 3). Moreover, we showed that both the use of two complementary measures of signal irregularity (entropy and statistical complexity) and the multiscale approach could be critical to really uncover the spatio-temporal structure underlying normal and altered perception. Interestingly, weighted measures of entropy (MWPE) and statistical complexity (MWJSC) were indeed more able to disassociate the brain signal underlying normal and altered perception with respect to their classical counterparts (MPE and MJSC), while LZC measures appeared less sensitive in this context. Another main finding of this study was that functional connectivity networks were largely perturbed by the artificial stimulation compared to the regular video. An average higher number of significant connections (GFC) in the gamma band was observed when participants watched the DD video, resulting in a higher interconnected connectivity pattern with respect to the OR video. This finding is comparable to the results of Roseman et al. [5], in which they tested resting state functional connectivity networks under the influence of psilocybin and methylenedioxymethamphetamine (MDMA). Crucially, they found a general increase in the between-network functional connectivity only induced by psilocybin, implying that these changes in functional connectivity could be exclusive to psychedelic drugs and related to their profound effects on perceptual phenomenology. Furthermore, consistent with our findings, Barnett et al. [13] found that the strongest increase in functional connectivity was observed in the gamma band. In addition, we also found that the AGE was higher in DD with respect to OR in the gamma band, a pattern similar to the findings of Viol et al. [31] in which a higher level of GE induced by the ingestion of Ayahuasca was observed. Taken together, these results are in line with the literature on actual psychedelic perceptual experiences, proving that the artificial stimulation elicited similar brain patterns. A possible explanation for why this artificial stimulation is so effective to simulate psychedelic perception is that DeepDream can be conceived as a generative process that imposes a strong perceptual prior on incoming sensory data [14], mimicking what is currently thought to be the computational process underlying perceptual inference [37]. Under this framework, known in the literature as Predictive Coding [38,39,40], another crucial finding of this study can be accounted. In the Predictive Coding framework, the brain is conceived as an inferential machine, making continuous predictions about the causes of the sensory data and optimizing its generative models of the world by computing prediction errors (discrepancies between data and expectations). From a functional point of view, it is thought that there is a fundamental asymmetry in the way these predictions and prediction errors are encoded and transmitted among the neural circuitry [41]. Broadly speaking, bottom-up prediction errors are conveyed at a higher frequency (e.g., gamma), while top-down predictions are transmitted at slower frequencies (e.g., alpha and beta) [42]. According to this framework, our finding about the increase in functional connectivity only in the gamma band could be interpreted as the neural system’s overload of prediction error messages passing due to the highly unpredictable sensory data it gathered. Another theoretical framework in which our findings can be enlightened is the entropic brain hypothesis [3], in which the brain is conceived as a non-linear far-from-equilibrium thermodynamical system. In this framework, during psychedelic experiences, the brain operates at a criticality state, where it has access to a larger repertoire of physical states and therefore has a higher level of entropy. In contrast, under normal waking consciousness, the brain is supposed to be in a regime of sub-criticality, since this entropy suppression allows the emergence of metacognitive functions, such as reality-testing and self-awareness. Our findings also conform to these theoretical predictions, by adding to previous results the evidence that also simulated hallucinations can lead the brain to a highly entropic criticality state. Some interesting future directions could be the use of virtual reality (VR) to present the stimuli to participants (a method called the Hallucination Machine in Suzuki et al. [14]) while collecting EEG. This would allow a higher level of engagement and ecological validity to the stimulation by enhancing participants’ sense of presence. Another direction could be to enlarge the stimulus set by exploring the parameter space of DeepDream, for example by generating multiple videos that are optimized to match sequentially from low-level to high-level activation layers. This would shed light on how the low-level features modulated the brain dynamics, since in this study we focused more on the semantic features of the stimulation. Moreover, the careful investigation of how and to what extent DeepDream can be compared to actual altered experience would have a considerable impact on the field of psychedelic research, especially in light of recent findings suggesting an important role of the external stimulation on the brain dynamics of hallucinatory states [43]. Since DeepDream, by definition, mimics hallucinatory perception using external stimulation, it would be interesting to use it as a tool to further explore this aspect of psychedelic brain dynamics. We also encourage future studies to directly compare DeepDream with actual psychedelic experience and to investigate other aspects of the brain dynamics relative to psychedelic states such as the alpha suppression [44] and directed functional connectivity [13]. In conclusion, we demonstrated with neuroimaging evidence how the use of modern deep learning algorithms could furnish psychedelic research with a new tool to study altered perception.

## Data Availability

The data presented in this study are available upon reasonable request from the corresponding author.

## Supplementary Materials

The following are available online at https://www.mdpi.com/article/10.3390/e23070839/s1. Figure S1: (**A**) MPE values for DD and OR along the time scales in the frontal, temporo-parietal and occipital ROI. Shaded areas represent standard error of the mean. Gray lines indicate statistical significance (*p* < 0.05, cluster corrected). (**B**) MJSC values for DD and OR along the time scales in the frontal, temporo-parietal and occipital ROI. Shaded areas represent standard error of the mean. Gray lines indicate statistical significance (*p* < 0.05, cluster corrected). (**C**) Topographic maps depicting the MPE of DD and OR in the significant time scale range 6–16. (**D**) Topographic maps depicting the MJSC of DD and OR in the significant time scale range 7–17. Figure S2: (**A**) pLZC values for DD and OR along the time scales in the frontal, temporo-parietal and occipital ROI. Shaded areas represent standard error of the mean. (**B**) mLZC values for DD and OR along the time scales in the frontal, temporo-parietal and occipital ROI. Shaded areas represent standard error of the mean.
