# Supplementary material for: Increased Entropic Brain Dynamics during DeepDream-Induced Altered Perceptual Phenomenology
Source: Entropy (Basel). 2021 Jun 30;23(7):839. doi: 10.3390/e23070839 (PMC8306862; doi:10.3390/e23070839)
Supplement: Supplementary file 1 [file entropy-23-00839-s001.zip › entropy-1221252-supplementary.pdf]

Supplementary Material

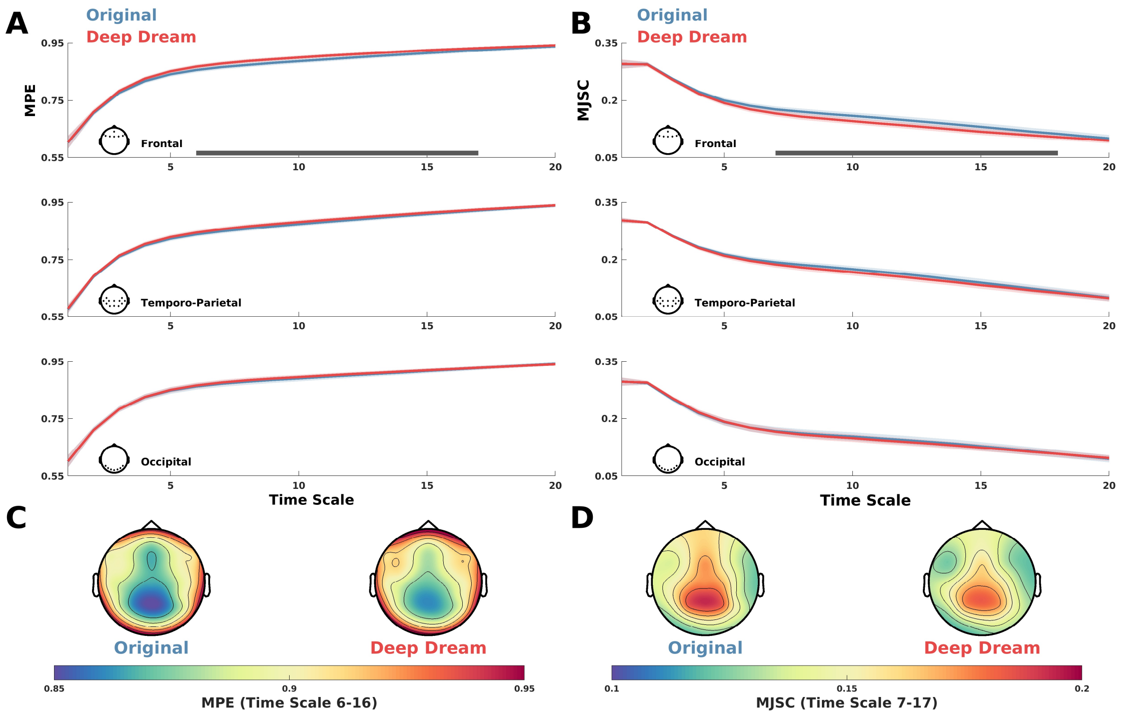

Figure S1. (**A**) MPE values for DD and OR along the time scales in the frontal, temporo-parietal and occipital ROI. Shaded areas represent standard error of the mean. Gray lines indicate statistical significance ($p < 0.05$, cluster corrected). (**B**) MJSC values for DD and OR along the time scales in the frontal, temporo-parietal and occipital ROI. Shaded areas represent standard error of the mean. Gray lines indicate statistical significance ($p < 0.05$, cluster corrected). (**C**) Topographic maps depicting the MPE of DD and OR in the significant time scale range 6–16. (**D**) Topographic maps depicting the MJSC of DD and OR in the significant time scale range 7–17.

Copyright: © 2021 by the authors. Licensee MDPI, Basel, Switzerland. This article is an open access article distributed under the terms and conditions of the Creative Commons Attribution (CC BY) license (http://creativecommons.org/licenses/by/4.0/).

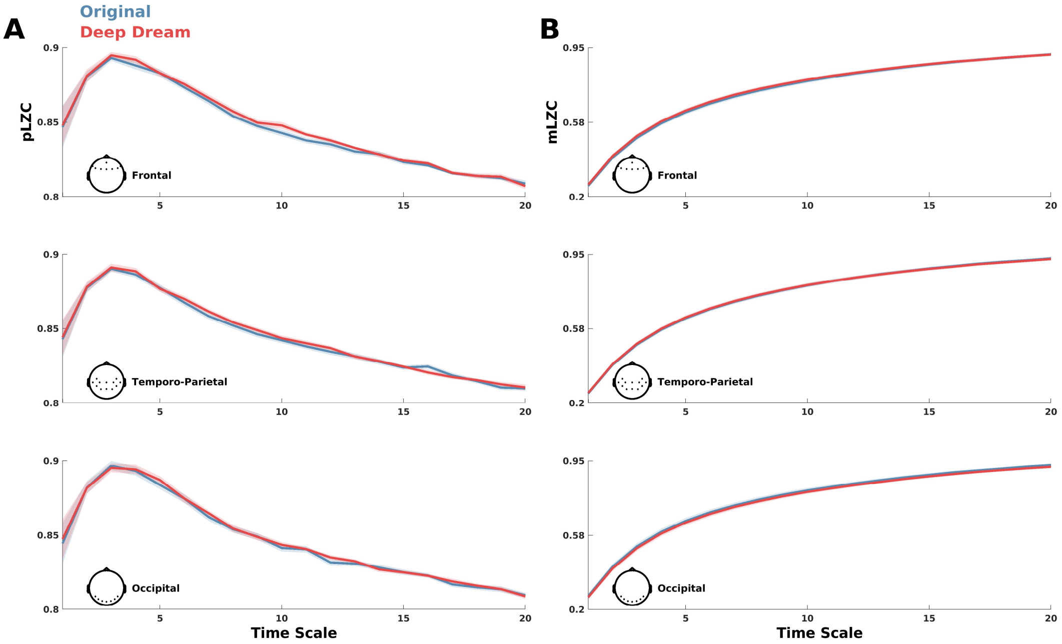

Figure S2. (**A**) pLZC values for DD and OR along the time scales in the frontal, temporo-parietal and occipital ROI. Shaded areas represent standard error of the mean. (**B**) mLZC values for DD and OR along the time scales in the frontal, temporo-parietal and occipital ROI. Shaded areas represent standard error of the mean.
